# HTLV-1 associated myelopathy of rapid onset and progression following living-donor kidney transplant: Clinical description and initial response to empirical treatment

**DOI:** 10.1186/1742-4690-8-S1-A56

**Published:** 2011-06-06

**Authors:** Liliana Gonzales, Jorge L Alave, Mario Paredes, Carlos Molina, Giovanni Lopez, Carolina Alvarez, Martin Tipismana, Eduardo Gotuzzo

**Affiliations:** 1 Departamento de Trasplante Renal, Hospital Nacional Guillermo Almenara Irigoyen, Lima, Perú; 2 Instituto de Medicina Tropical “Alexander von Humboldt”, Universidad Peruana Cayetano Heredia, Lima, Perú; 3 Instituto de Medicina Tropical “Alexander von Humboldt”, Universidad Peruana Cayetano Heredia, Lima, Perú; 4 Departamento de Enfermedades Infecciosas, Tropicales y Dermatológicas, Hospital Nacional Cayetano Heredia, Lima, Perú

## Objective and methods

To describe a case of HTLV-1 associated myelopathy (HAM/TSP) of rapid onset and progression in a renal transplant recipient, and the initial response to empirical treatment.

## Results

A 50-year-old man was admitted with 12 weeks of evolving spastic paraparesis and urinary–fecal retention, five months after a successful living-donor renal transplantation due to end-stage chronic kidney disease. He was on basiliximab, metilprednisolone, tacrolimus, mychophenolate and prednisone for immunosupression.

The physical examination showed pyramidal syndrome predominantly in lower limbs, with hyperalgesia and hypersthesia. The Kurtzke's Expanded Disability Scale (EDSS) showed a score of 7.5. Magnetic resonance imaging showed enlargement and inflammatory pattern in whole spinal cord. HTLV-1 ELISA was positive both in fresh blood and cerebrospinal fluid (CSF). HTLV-1 proviral load by Sybr-Green quantitative real-time PCR performed twice were 1879 copies/10,000 peripheral blood lymphocytes, and 6465 copies/10,000 CSF lymphocytes. Although HTLV-1 screening had not been performed before transplantation neither in the donor nor in the recipient, results were positive in a fresh blood sample from the donor, and negative in one from the recipient cryopreserved two years before transplantation.

The patient received empirical treatment with a pulse of corticosteroids, zidovudine, lamivudine, prednisone and baclofen. After one month of treatment, the EDSS showed a score of 7.

## Conclusion and interpretations

This case report shows a very acute clinical presentation of HAM/TSP with extensive and diffuse spinal cord involvement in an immunosuppressed patient. In endemic areas, HTLV-1 screening should be included in protocols of organs’ donors and recipients before transplantation.

